# CO_2_ Solubility in Aqueous Solutions of Amine–Ionic Liquid Blends: Experimental Data for Mixtures with AMP and MAPA and Modeling with the Modified Kent–Eisenberg Model

**DOI:** 10.3390/molecules30183832

**Published:** 2025-09-21

**Authors:** Giannis Kontos, Ioannis Tsivintzelis

**Affiliations:** Department of Chemical Engineering, Aristotle University of Thessaloniki, 54124 Thessaloniki, Greece

**Keywords:** CO_2_ capture, ionic liquids, choline glycine, 1-butyl-3-methylimidazolium hydrogen sulfate, AMP, MAPA, Kent–Eisenberg model, ionic liquid–amine blends

## Abstract

Carbon dioxide (CO_2_) capture using alkanolamines remains the most mature technology, yet faces challenges including solvent loss, high regeneration energy and equipment corrosion. Ionic liquids (ILs) are proposed as alternatives, but their high viscosity and production costs hinder industrial use. Thus, blending ILs with amines offers a promising approach. This work presents new experimental data for aqueous blends of 1-butyl-3-methylimidazolium hydrogen sulfate, $\left[Bmim^{+}\right]\left[HSO_{4}^{-}\right],$ with 2-amino-2-methyl-1-propanol (AMP) and 3-(methylamino)propylamine (MAPA) and for choline glycine, $\left[Ch^{+}\right]\left[Gly^{-}\right]$, with AMP, modeled using the modified Kent–Eisenberg approach. It was shown that substituting a portion of the amine with $\left[Bmim^{+}\right]\left[HSO_{4}^{-}\right]$ reduces CO_2_ uptake per mole of amine due to the lower solution’s basicity, despite the added sites for physical absorption. In contrast, the replacement of an amine portion with $\left[Ch^{+}\right]\left[Gly^{-}\right]$ enhances both physical and chemical interactions, leading to increased CO_2_ solubility per mole of amine. Finally, replacing a small portion of water with $\left[Ch^{+}][Gly^{-}\right]$ does not significantly alter the bulk CO_2_ solubility (moles of CO_2_ per kg of solvent) but lowers the solvent’s vapor pressure. Given the non-toxic nature of $\left[Ch^{+}][Gly^{-}\right]$, the resulting solvent poses no added environmental risk. Model predictions agree well with experimental data (deviations of 2.0–11.6%) and indicate low unreacted amine content at CO_2_ partial pressures of 1–10 kPa for carbamate-forming amines, i.e., $\left[Gly^{-}\right]$, and MAPA. Consequently, at higher CO_2_ partial pressures, the solubility increases due to carbamate hydrolysis and molecular CO_2_ dissolution.

## 1. Introduction

Acid gases, primarily CO_2_ and H_2_S, are found in various industrial gas streams, including natural gas (NG), syn-gas and refinery gases [1]. Their removal is critical both economically and environmentally due to their detrimental effects on equipment integrity, product specifications [2], on the one hand, and on climate change, and acid precipitation [3], on the other hand. Corrosion of pipelines and storage equipment is largely attributed to the presence of these gases [4].

H_2_S, in particular, is highly toxic and deactivates refinery catalysts [5]. From the environmental point of view, H_2_S, in the presence of oxygen at high temperatures, yields sulfur oxides, which contribute to greenhouse gas emissions and acid rain [6].

CO_2_ concentration exceeds 2% in more than half of the world’s natural gas (NG) reserves [3]. CO_2_ needs to be separated from NG because it has no calorific (heating) value, and consequently, acts as a diluent, reducing the energy content of NG and increasing the transportation and storage costs [1]. Additionally, CO_2_ must be removed during ammonia synthesis to prevent catalyst poisoning [1]. Since the beginning of industrialization, global atmospheric CO_2_ has been dramatically increasing, primarily due to fossil fuel combustion, resulting in a temperature increase during the 20th century [7]. However, fossil fuels remain an important source of energy, with this figure set to remain so for the next few decades [7], if urgent measures are not taken. Carbon capture and storage or utilization technologies are a valuable option to mitigate the consequences.

Among the available carbon capture and storage (CCS) technologies, post-combustion CO_2_ capture using alkanolamines appears to be the most mature technology [1,3]. However, this technology shows a number of drawbacks, including solvent losses [8], high regeneration energy requirements [9] and equipment corrosion [10]. Consequently, numerous new solvents were recently presented, including ionic liquids (ILs).

ILs are defined as organic salts that exist in a liquid state at easily accessible temperatures, usually below 373K [11]. Most often, they are composed of a large, bulky, and asymmetric organic cation, such as imidazolium or pyridinium, and an anion selected from a wide variety of anions, such as hexafluorophosphate ${\left[{Pf}_{6}\right]}^{-}$ or trifluoromethanesulfonate, ${\left[TfO\right]}^{-}$ [12], as shown in Table 1.

Ionic liquids (ILs) have the potential to replace organic solvents, particularly volatile ones, since volatile organic compounds (VOCs) are a major contributor to total industrial atmospheric emissions and, consequently, to environmental pollution [13]. ILs are considered environmentally benign alternatives to VOCs due to their unique properties, such as very low and negligible vapor pressure [14,15], low or non-flammability [16] and high thermal stability [17,18], which enable their use across a wide range of temperatures [19]. Certain industrial processes, such as the Biphasic Acid Scavenging Utilizing Ionic Liquids (BASIL) process [20], already use ionic liquids as solvents.

In 2001, ILs were proposed for the first time as alternative solvents for CO_2_ absorption [21]. Since then, extensive research has focused on this application [19]. However, several significant limitations hinder the widespread industrial adoption of ILs, including their high viscosity [22] and the high IL synthesis cost [19]. Moreover, some ILs are toxic [23] and/or non-biodegradable [24], raising health and environmental concerns.

Blending of ILs with amines has attracted considerable research interest as an alternative approach to CO_2_ capture, aiming to overcome the drawbacks that each class of fluids exhibits when used independently. Consequently, several studies on kinetics [25,26], regeneration performance [27] and CO_2_ solubility have appeared in the literature. In more detail, Yuan et al. studied blends of choline glycine IL, $\left[Ch^{+}][Gly^{-}\right]$, with *N*-methyl-diethanolamine (MDEA). They reported that although CO_2_ loading of $\left[Ch^{+}][Gly^{-}\right]$ (x = 5, 10, 15, 20 wt.%)-MDEA[(30 − x) wt.%] aqueous solutions decreased with increasing IL content, the absorption rate constant of all blends was higher than that of the aqueous 30 wt.% MDEA solution [28]. In the same study, the viscosity of the aqueous blends was significantly lower than that of their individual components (43.9 and 84.3 mPa∙s, at 308.2 K, for pure $\left[Ch^{+}][Gly^{-}\right]$ and MDEA, respectively). In another study, the addition of 10 wt.% 1-butyl-3-methylimidazolium acetate, $\left[Bmim^{+}\right]\left[Ac^{-}\right],$ in 30 wt.% MDEA aqueous solution, at 10 bar and ambient temperature, increased the CO_2_ solubility by 53.92% compared to that of the 30 wt.% aqueous MDEA solution [29]. Finally, the addition of 1-hexyl-3-methylimidazolium glycinate, [Hmim^+^][Gly^−^], in aqueous solutions of 2-amino-2-methyl-1-propanol (AMP), significantly enhanced the CO_2_ reactivity [30].

**Table 1:** Some commonly used cations and anions to form ionic liquids.

CATIONS		ANIONS	
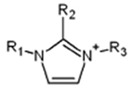	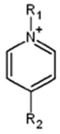	Br^−^, Cl^−^, I^−^	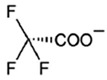
Imidazolium	Pyridinium	Bromide, chloride, iodide	$\mathrm{Trifluoroacetate},\;{\left[TfA\right]}^{-}$
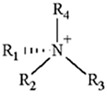	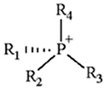	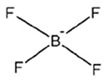	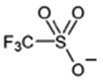
Ammonium	Phosphonium	$\mathrm{Tetrafluoroborate},\;{\left[Bf_{4}\right]}^{-}$	$\mathrm{Trifluoromethanesulfonate},\;{\left[TfO\right]}^{-}$
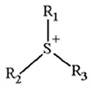	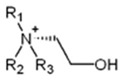	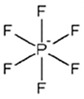	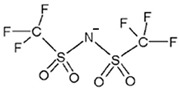
Sulfonium	Cholinium	Hexafluorophosphate, [Pf_6_]^−^	$\mathrm{Bis}(\mathrm{trifluoromethylsulfonyl})\mathrm{imide},\;{\left[Tf_{2}N\right]}^{-}$

In general, imidazolium-based ILs show relatively high physical CO_2_ absorption capacity and high CO_2_/N_2_ and CO_2_/H_2_ selectivity [31]. The interaction of CO_2_ (Lewis acid, LA) with the anion of ILs (Lewis base, LB) significantly affects CO_2_ solubility in ILs. The 1-ethyl-3-methylimidazolium hydrogen sulfate, $\left[Emim^{+}\right]\left[HSO_{4}^{-}\right]$, exhibits relatively high CO_2_ solubility presumably due to the high negative charge of oxygen atoms in the $\left[HSO_{4}^{-}\right]$ anion, resulting in stronger interactions with the partially positive charge of the carbon atom in CO_2_ [32]. Also, according to Raveendran and Wallen [33], the S=O bond becomes highly polarized when the sulfonyl group interacts with CO_2_. Moreover, $\left[Bmim^{+}\right]\left[HSO_{4}^{-}\right]$, due to the longer alkyl chain in its cation compared to $\left[Emim^{+}\right]\left[HSO_{4}^{-}\right]$, generally exhibits a larger free volume, and this structural feature is expected to enhance its affinity for CO_2_ molecules.

However, since the chemical absorption of CO_2_ is significantly higher than absorption resulting solely from physical interactions, various ILs with amine groups in their structure have been proposed, such as amino acid-based ionic liquids. The stoichiometric CO_2_ loading on an amino acid ionic liquid (AAIL) depends on the number and the type (sterically hindered or not) of amino groups in the amino acid anion. In general, non-hindered primary and secondary amine groups react with CO_2_ (in aqueous solutions) in a 1:2 stoichiometry (0.5 moles of CO_2_ per mole of amine group) through the carbamate formation mechanism, while hindered ones may follow the 1:1 stoichiometry (1 mole of CO_2_ per mole of amine group). Nevertheless, sometimes different reaction mechanisms occur, showing deviations from the above limits, i.e., a lysine-based IL, with two amino groups in lysine, was found to follow the 2:1 absorption ratio (2 moles of CO_2_ per mole of IL) [34].

In this work, $\left[Bmim^{+}\right]\left[HSO_{4}^{-}\right]$ and $\left[Ch^{+}][Gly^{-}\right]$ were chosen as additives to the aqueous solutions of 2-amino-2-methyl-1-propanol (AMP) and 3-(methylamino)propylamine (MAPA). The former IL, i.e., $\left[Bmim^{+}\right]\left[HSO_{4}^{-}\right]$, is expected to enhance physical CO_2_ absorption and the latter, i.e., $\left[Ch^{+}][Gly^{-}\right]$, to mainly enhance chemical CO_2_ absorption (Table 2). Moreover, both counterpart ions in choline–glycine amino acid ILs are non-toxic [35] and biodegradable [36]. In contrast to other ILs, which are extremely viscous, $\left[Ch^{+}][Gly^{-}\right]$ exhibits lower viscosity; thus, CO_2_ solubility suppression due to viscosity effect is not expected. Furthermore, AMP is a relatively less corrosive [10] and thermally stable amine [37]. Due to its steric hindrance, it exhibits an equimolar CO_2_ loading (1 mole of CO_2_ per mole of amine) [38]. MAPA is a biodegradable diamine with high CO_2_ solubility due to the presence of two functional groups, a primary and a secondary amine group [38]. Due to its high corrosiveness [39], the better way to use MAPA is as a CO_2_ absorption promoter in solvent blends. Although aqueous blends of $\left[Bmim^{+}\right]\left[HSO_{4}^{-}\right]$-AMP, $\left[Bmim^{+}\right]\left[HSO_{4}^{-}\right]$-MAPA and $\left[Ch^{+}][Gly^{-}\right]$-AMP show potential for high CO_2_ absorption performance, CO_2_ solubility in these blends has not yet been studied in the literature.

**Table 2:** Structure of ILs and amines used for the preparation of IL + amine aqueous solutions.

IONIC LIQUIDS	
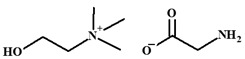	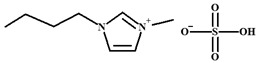
Choline glycine, [Ch^+^][Gly^−^]	$1\text{-}\mathrm{Butyl}\text{-}3\text{-}\mathrm{methylimidazolium}\;\mathrm{hydrogen}\;\mathrm{sulfate},\;{[Bmim}^{+}]$$[{HSO}_{4}^{-}$]
**AMINES**	
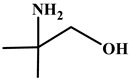	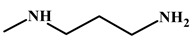
2-amino-2-methyl-1-propanol, AMP	3-(methylamino)propylamine, MAPA

## 2. Materials and Methods

### 2.1. Materials

Pressurized CO_2_ (99.9% vol) was purchased from Air Liquide. Choline glycine, $\left[Ch^{+}][Gly^{-}\right]$, was synthesized according to a method previously described and the purity was checked by ^1^H and ^3^C NMR [40]. 1-Butyl-3-methylimidazolium hydrogen sulfate, $\left[Bmim^{+}\right]\left[HSO_{4}^{-}\right]$, purity > 94.5 wt.%, was procured from Aldrich and used as received. 2-Amino-2-methyl-1-Propanol, AMP (Aldrich, St. Louis, MO, USA), and 3-(methylamino)propylamine, MAPA (Sigma-Aldrich), with purity higher than 99 wt.%, were used as received without further purification. For preparing aqueous IL-amine solutions, distilled water (HPLC) provided by the Chem lab was used.

### 2.2. Experimental Apparatus and Procedure

**Figure 1:**
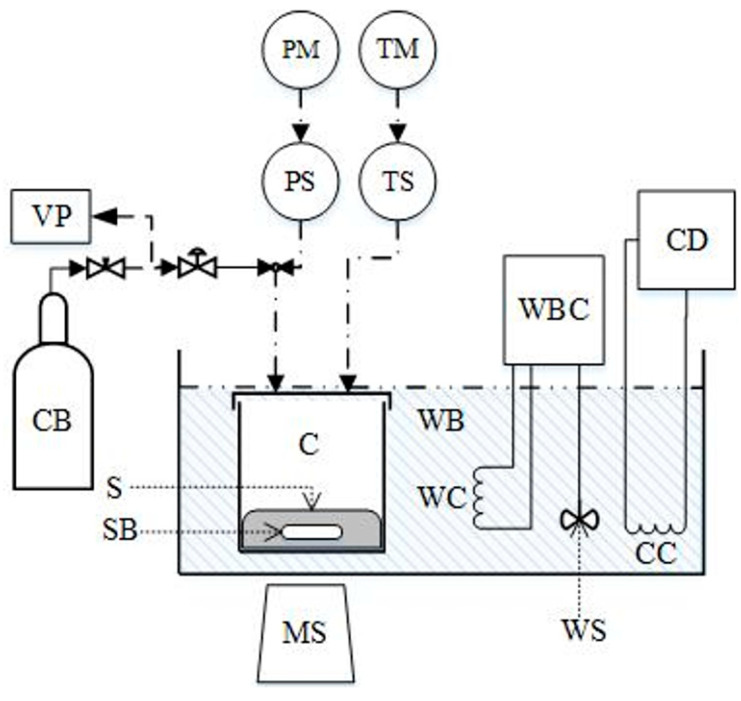
Experimental apparatus [41] where *C* stands for the equilibrium cell, *CB* for the CO_2_ flask, *CC* for cooling coil, *CD* for cooling device, *MS* for magnetic stirrer, *PM* for pressure indicator, *PS* for pressure sensor, *S* for solution, *SB* for stirring bar, *TM* for temperature indicator, *TS* for Pt-100 thermometer, *WB* for water bath, *WBC* for temperature controller/heater, *WC* for heating coil, *WS* for stirrer, *VP* vacuum pump.

The used apparatus (Figure 1) and procedure for performing the solubility experiments, as well as the validation method, were detailed in our previous work [41] and are briefly revisited here. CO_2_ solubility in aqueous IL-amine mixtures was determined using a high-pressure stainless-steel equilibrium cell with an internal volume of 152.2 ± 1.6 cm^3^ (volume measured at 25 °C). The setup included a WIKA A-10 pressure transmitter (±0.5%) and a Pt-100 thermometer (accuracy of ±0.01 K). The cell was submerged in a temperature-controlled water bath (Grant TC-120, stability ±0.1 K) to maintain isothermal conditions. A weighed quantity of the aqueous IL-amine solution (standard uncertainty of ±0.001 g) was loaded into the cell, followed by a measured amount of pure CO_2_ (weighed with standard uncertainty of ±0.005 g), and the system was heated to the target temperature. Before initiating measurements, the vessel was repeatedly evacuated to ensure the solvent remained under its own vapor pressure. At the beginning of each experiment, when CO_2_ is introduced into the cell, a high pressure is recorded, which decreases as CO_2_ is continuously absorbed by the liquid solution. When phase equilibrium is established, pressure remains stable. Equilibrium was considered achieved when pressure readings remained stable for a minimum of one hour at constant temperature. Throughout the experiment, the temperature and pressure values were were continuously recorded.

CO_2_ solubility, ${a_{CO}}_{2}$, is determined using the CO_2_ density, ${d_{CO}}_{2}$, [42], the density of the solvent, $d_{sol}$, the total mass of the solvent loaded in the cell, $m_{sol}$, the total moles of CO_2_ added in the equilibrium cell, ${n_{CO}}_{2}$, the volume of the cell $V_{T}$, the volume of the solvent $V_{sol}$ and the volume of the vapor phase, $V_{vap}$. It is expressed as the moles of CO_2_ absorbed in the liquid phase, $n_{CO_{2}}^{L}$, per moles of component *A*, $n_{A}$, as shown below:

(1) $$V_{sol}=\frac{m_{sol}}{d_{sol}}$$

(2) $$V_{vapor}\approx V_{T}-V_{sol}$$

(3) $$m_{CO_{2}}^{G}\approx V_{vap}\;{d_{CO}}_{2}$$

(4) $$n_{CO_{2}}^{L}=\frac{m_{CO_{2}}^{L}}{{{Mr}_{CO}}_{2}}\approx \frac{{m_{CO}}_{2}-m_{CO_{2}}^{G}}{{{Mr}_{CO}}_{2}}$$

(5) $$w_{A}=\frac{m_{A}}{m_{sol}}$$

(6) $$n_{A}=\frac{m_{A}}{{Mr}_{A}}=\frac{w_{A}m_{sol}}{{Mr}_{A}}$$

(7) $${a_{CO}}_{2}=\frac{n_{CO_{2}}^{L}}{n_{A}}$$

In Equations (1)–(7), *m* stands for the mass, *w* for the weight fraction, *V* for the volume, *n* for the number of moles, *Mr* for the molecular weight and *d* for the density. Superscripts *G* and *L* stand for gas and liquid, respectively, while subscripts *sol*, *vapor*, *T*, *A* and CO_2_ stand for solvent, vapor phase, total, component *A* and CO_2_ compound, respectively.

The CO_2_ partial pressure is estimated by subtracting the solution’s vapor pressure (estimated using Raoult’s law) from the total measured pressure. Given the very low vapor pressure of the solvent, this approximation introduces small corrections at relatively high pressures (above 100 kPa), while it is widely adopted in similar pressure decay experimental studies [38,43,44]. The change in the gas phase volume due to liquid expansion as it absorbs CO_2_ is assumed to be insignificant, owing to the low vapor phase density and the large total volume of the cell, in line with previous studies [45]. It is also assumed that the vapor pressure of the ionic liquid is very low and can be neglected. Therefore, only CO_2_, H_2_O and amine are considered present in the vapor phase.

The studied solutions were prepared by mixing amounts (weighted with an accuracy of 0.001 g) of ionic liquid, amine and water in a 100 mL volumetric flask (Din A) at room temperature. From this, the weight percent and density of each solution were calculated. Densities at 25, 40, 50 and 60 °C were similarly determined by accounting for flask volume changes at each temperature (estimated using HPLC-grade water). The compositions of the studied IL + Am aqueous solutions are shown in Table 3. 

In all cases, a 30 wt.% aqueous solution was investigated, containing 10 or 20 wt.% IL and 20 or 10 wt.%, respectively, amine. Such total concentration of active compounds (IL + amine) is typical for amine and alkanolamine solutions used in CO_2_ capture.

**Table 3:** Compositions of the studied aqueous solutions.

IL + Amine	Amine/IL (Molar Ratio)	IL ( wt.%)	Amine ( wt.%)
$\left[Bmim^{+}\right]\left[HSO_{4}^{-}\right]$ + AMP	5.32	9.93	19.94
$\left[Bmim^{+}\right]\left[HSO_{4}^{-}\right]$ + AMP	1.33	19.74	9.92
$\left[Bmim^{+}\right]\left[HSO_{4}^{-}\right]$ + MAPA	5.36	10.01	20.00
$\left[Ch^{+}][Gly^{-}\right]$ + AMP	4.00	10.03	20.04
$\left[Ch^{+}][Gly^{-}\right]$ + AMP	1.00	20.05	10.08

## 3. The Modified Kent–Eisenberg Model

For systems comprising CO_2_, a carbamate-forming amine ($R_{a}R_{b}NH$) and water, the chemical equilibrium can be described by the following reactions [46,47]:

(8) $${R_{a}R_{b}NH}_{2}^{+}\overset{{\;K}_{11}\;}{↔}R_{a}R_{b}NH{+H}^{+}\;$$

(9) $$H_{2}O+{CO}_{2}\overset{K_{2}}{↔}H^{+}+{HCO}_{3}^{-}$$

(10) $${HCO}_{3}^{-}\overset{K_{3}}{↔}H^{+}{+CO}_{3}^{-2}$$

(11) $$H_{2}O\;\overset{{\;K}_{4}}{↔}H^{+}+{OH}^{-}$$

(12) $$R_{a}R_{b}NCOO^{-}+H_{2}O\;\;\overset{{\;K}_{5}\;}{↔}{\;\;R}_{a}R_{b}NH+{HCO}_{3}^{-}\;\;$$

where ${R_{a}R_{b}NH}_{2}^{+}$ and $R_{a}R_{b}NCOO^{-}$ denote the protonated form of the amine and the carbamate anion, respectively. The equilibrium constants of reactions (8)–(12) can be written as follows:

(13) $$K_{11}=\frac{\left[R_{a}R_{b}NH\right]\left[H^{+}\right]}{\left[{R_{a}R_{b}NH}_{2}^{+}\right]}$$

(14) $$K_{2}=\frac{\left[{HCO}_{3}^{-}\right]\left[H^{+}\right]}{\left[CO_{2}\right]}$$

(15) $$K_{3}=\frac{\left[{CO}_{3}^{-2}\right]\left[H^{+}\right]}{\left[{HCO}_{3}^{-}\right]}$$

(16) $${K_{4}=[OH}^{-}]\left[H^{+}\right]$$

(17) $$K_{5}=\frac{\left[R_{a}R_{b}NH\right]\left[{HCO}_{3}^{-}\right]}{\left[R_{a}R_{b}NCOO^{-}\right]}$$

The molecular CO_2_ concentration in the liquid phase is determined by Henry’s law, expressed as follows:

(18) $$P_{{CO}_{2}}=H_{{CO}_{2}}\left[{CO}_{2}\right]$$

where $H_{CO2}$ and $P_{CO2}$ stand for Henry’s constant and CO_2_ partial pressure, respectively.

The overall mass and charge balance equations can be written as follows:

(19) $${\left[R_{a}R_{b}NH\right]}_{t}=\left[R_{a}R_{b}NH\right]+\left[{R_{a}R_{b}NH}_{2}^{+}\right]+\left[R_{a}R_{b}NCOO^{-}\right]$$

(20) $$\alpha_{CO_{2}}{\left[R_{a}R_{b}NH\right]}_{t}=\left[R_{a}R_{b}NCOO^{-}\right]+\left[{HCO}_{3}^{-}\right]+\left[{CO}_{3}^{-2}\right]+\left[{CO}_{2}\right]$$

(21) $$\left[H^{+}\right]+\left[{R_{a}R_{b}NH}_{2}^{+}\right]=\left[R_{a}R_{b}NCOO^{-}\right]+\left[{HCO}_{3}^{-}\right]+2\left[{CO}_{3}^{-2}\right]+{[OH}^{-}]$$

where $\alpha_{CO_{2}}$ denotes the CO_2_ loading in the solution (expressed as moles of CO_2_ per mole of amine) and ${\left[R_{a}R_{b}NH\right]}_{t}$ denotes the total amine concentration in the mixture.

According to Kontos et al. [38], Equation (13) through (21) simplify to a system of two equations with two unknowns, i.e., $\left[H^{+}\right]$ and $\left[{R_{a}R_{b}NH}_{2}^{+}\right]$,

(22) $${\left[R_{a}R_{b}NH\right]}_{t}=\left(1+\frac{K_{11}}{\left[H^{+}\right]}+\frac{K_{11}K_{2}\left[{CO}_{2}\right]}{K_{5}{\left[H^{+}\right]}^{2}}\right)\left[{R_{a}R_{b}NH}_{2}^{+}\right]$$

(23) $$\left[{R_{a}R_{b}NH}_{2}^{+}\right]\left(1-\frac{K_{11}K_{2}\left[{CO}_{2}\right]}{K_{5}{\left[H^{+}\right]}^{2}}\right)=\frac{K_{4}}{\left[H^{+}\right]}+\frac{K_{2}\left[{CO}_{2}\right]}{\left[H^{+}\right]}+\frac{{2K}_{2}K_{3}\left[{CO}_{2}\right]}{{\left[H^{+}\right]}^{2}}-\left[H^{+}\right]$$

and a polynomial equation is obtained as follows:

(24) $$A{\left[H^{+}\right]}^{5}+B{\left[H^{+}\right]}^{4}+C{\left[H^{+}\right]}^{3}+D{\left[H^{+}\right]}^{2}+E\left[H^{+}\right]+F=0$$

where the relations for parameters *A*, *B*, *C*, *D*, *E* and *F* can be found in the literature [38,48]. The CO_2_ loading is subsequently calculated using the following expression:

(25) $$\alpha_{CO_{2}}=\frac{\left(\left[CO_{2}\right]+\frac{K_{2}\left[CO_{2}\right]}{\left[H^{+}\right]}+\frac{K_{2}K_{3}\left[CO_{2}\right]}{{\left[H^{+}\right]}^{2}}+\left[RRNCOO^{-}\right]\right)}{{\left[R_{a}R_{b}NH\right]}_{t}}$$

where

(26) $$\left[R_{a}R_{b}NCOO^{-}\right]=\frac{K_{11}K_{2}\left[CO_{2}\right]{\left[R_{a}R_{b}NH\right]}_{t}}{\left(K_{5}{\left[H^{+}\right]}^{2}+K_{11}K_{5}\left[H^{+}\right]+K_{11}K_{2}\left[CO_{2}\right]\right)}$$

For AMP solutions, no stable carbamates are formed, so reaction (12) can be neglected. Thus, $\left[R_{a}R_{b}NCOO^{-}\right]$ can be omitted in material and charge balances (Equations (19)–(21)), resulting in the following fourth-order polynomial equation:

(27) $$A{\left[H^{+}\right]}^{4}+B{\left[H^{+}\right]}^{3}+C{\left[H^{+}\right]}^{2}+D\left[H^{+}\right]+E=0$$

where the relations for the parameters *A*, *B*, *C*, *D* and *E* can be found in the literature [38,48]. Consequently, the total CO_2_ loading ($\alpha_{CO_{2}}$) can be calculated through the following equation:

(28) $$\alpha_{CO_{2}}=\frac{\left[CO_{2}\right]+\frac{K_{2}\left[CO_{2}\right]}{\left[H^{+}\right]}+\frac{K_{2}K_{3}\left[CO_{2}\right]}{{\left[H^{+}\right]}^{2}}}{{\left[R_{a}R_{b}NH\right]}_{t}}$$

In a mixture of one carbamate-forming ($R_{a}R_{b}NH$) and one non-carbamate-forming ($R_{c}R_{d}NH$) amine we need to consider the protonation of the non-carbamate-forming amine and the corresponding equilibrium constant:

(29) $${R_{c}R_{d}NH}_{2}^{+}\overset{K_{12}}{↔}R_{c}R_{d}NH{+H}^{+}$$

(30) $$K_{12}=\frac{\left[R_{c}R_{d}NH\right]\left[H^{+}\right]}{\left[{R_{c}R_{d}NH}_{2}^{+}\right]}$$

According to Kontos et al. [44], the material and charge balances for the mixture can be reduced to a single sixth-order polynomial equation in terms of the concentrations of hydrogen ions, $\left[H^{+}\right]$:

(31) $$A{\left[H^{+}\right]}^{6}+B{\left[H^{+}\right]}^{5}+C{\left[H^{+}\right]}^{4}+D{\left[H^{+}\right]}^{3}+E{\left[H^{+}\right]}^{2}+F\left[H^{+}\right]+G=0$$

where the relations for the parameters *A*, *B*, *C*, *D*, *E*, *F* and *G* can be found in Kontos et al. [44]. Then, the CO_2_ loading is calculated through the following relation [44]:

(32) $$\alpha_{CO_{2}}=\frac{\left(\left[CO_{2}\right]+\frac{K_{2}\left[CO_{2}\right]}{\left[H^{+}\right]}+\frac{K_{2}K_{3}\left[CO_{2}\right]}{{\left[H^{+}\right]}^{2}}+\left[R_{a}R_{b}NCOO^{-}\right]\right)}{{\left[R_{a}R_{b}NH\right]}_{t}+{\left[R_{c}R_{d}NH\right]}_{t}}$$

where

(33) $$\left[R_{a}R_{b}NCOO^{-}\right]=\frac{K_{11}K_{2}\left[CO_{2}\right]{\left[R_{a}R_{b}NH\right]}_{t}}{\left(K_{5}{\left[H^{+}\right]}^{2}+K_{11}K_{5}\left[H^{+}\right]+K_{11}K_{2}\left[CO_{2}\right]\right)}$$

In all cases, the temperature dependence of the equilibrium constants, $K_{i}$, and the Henry’s law constant is described by the following empirical relation [49]:

(34) $$\ln K_{i}=\frac{A_{i}}{T}+B_{i}lnT+C_{i}T+D_{i}$$

## 4. Results and Discussion

### 4.1. Experimental Solubility Results

**Figure 2:**
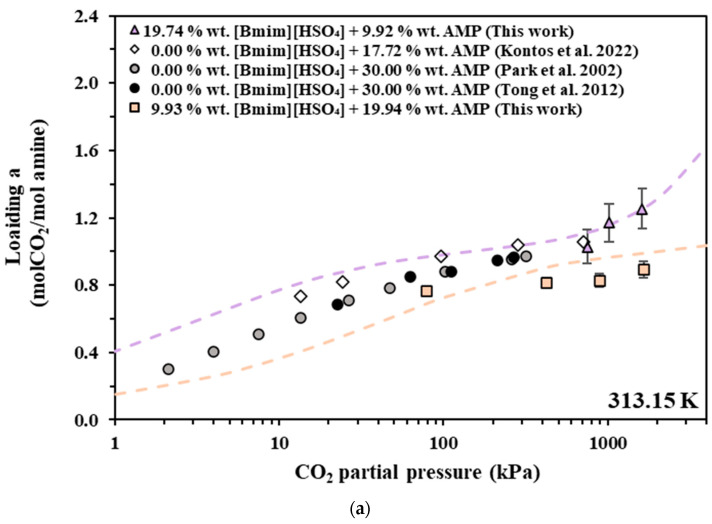

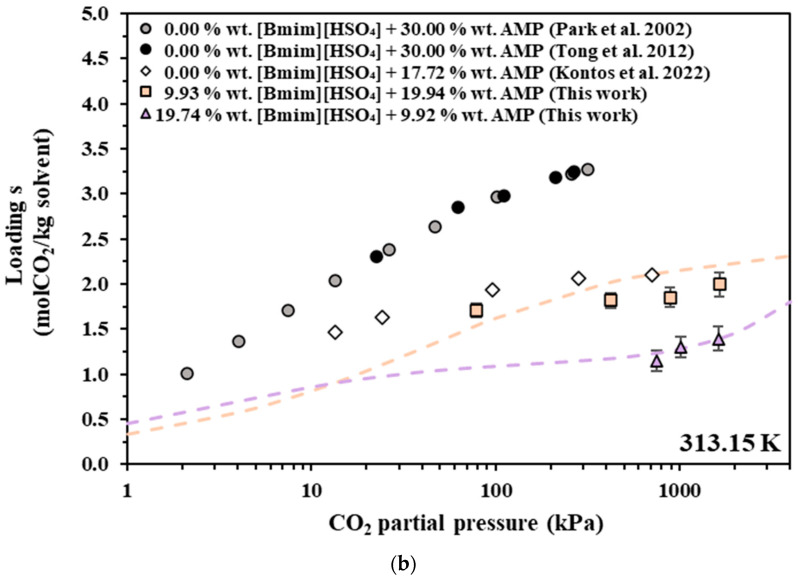
CO_2_ solubility in aqueous $\left[Bmim^{+}][HSO_{4}^{-}\right]$ + AMP (squares and triangles), AMP 17.72 wt.% (rhombus) [38] and AMP 30.00 wt.% (circles) [50,51] solutions at 313.15 K. Experimental data (squares and triangles) and modified Kent–Eisenberg correlations (dashed lines) for the solubility in $\left[Bmim^{+}][HSO_{4}^{-}\right]$ + AMP aqueous solutions expressed as (**a**) moles of CO_2_ per mole of amine and (**b**) as moles of CO_2_ per kg of solvent.

**Figure 3:**
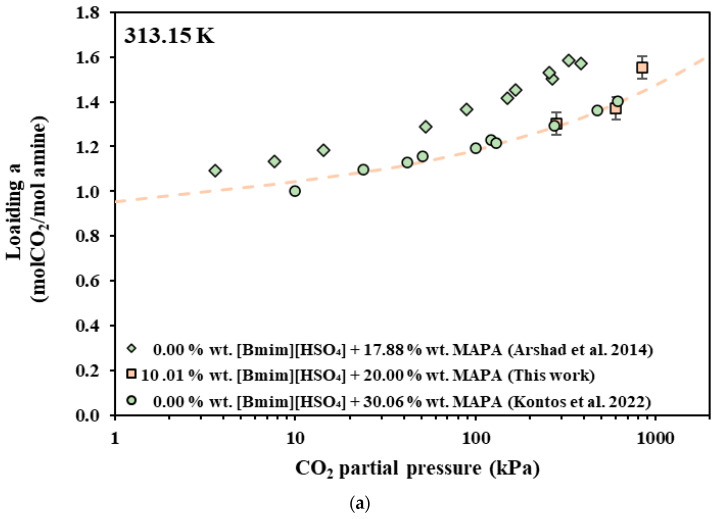

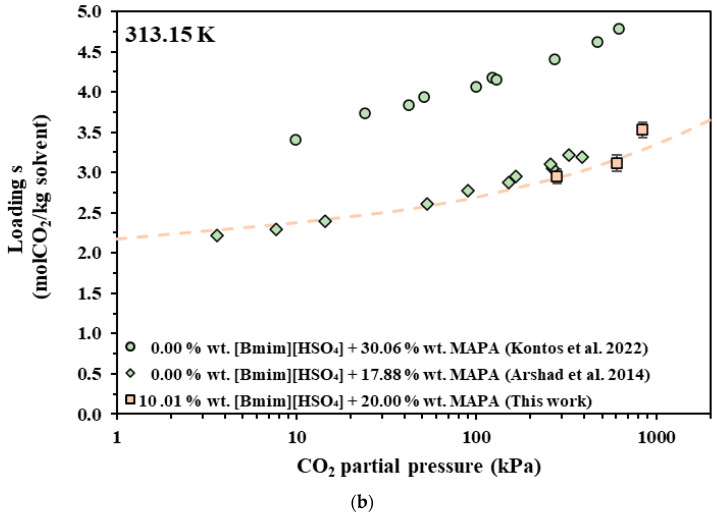
CO_2_ solubility in aqueous $\left[Bmim^{+}][HSO_{4}^{-}\right]$ + MAPA (squares), MAPA 17.88 wt.% (rhombus) [52] and MAPA 30.06 wt.% (circles) [38] solutions at 313.15 K. Experimental data (squares) and modified Kent–Eisenberg correlations (dashed line) for the solubility in $\left[Bmim^{+}][HSO_{4}^{-}\right]$ + MAPA aqueous solutions expressed as (**a**) moles of CO_2_ per mole of amine and (**b**) as moles of CO_2_ per kg of solvent.

**Figure 4:**
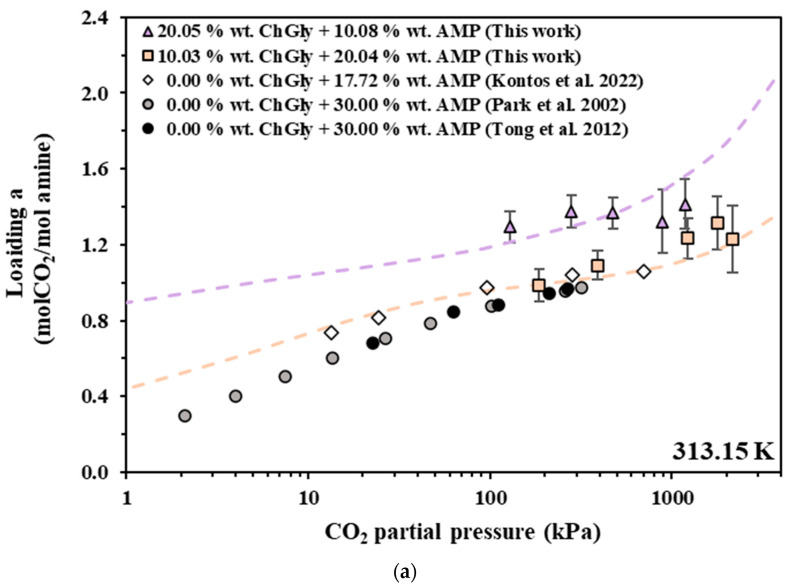

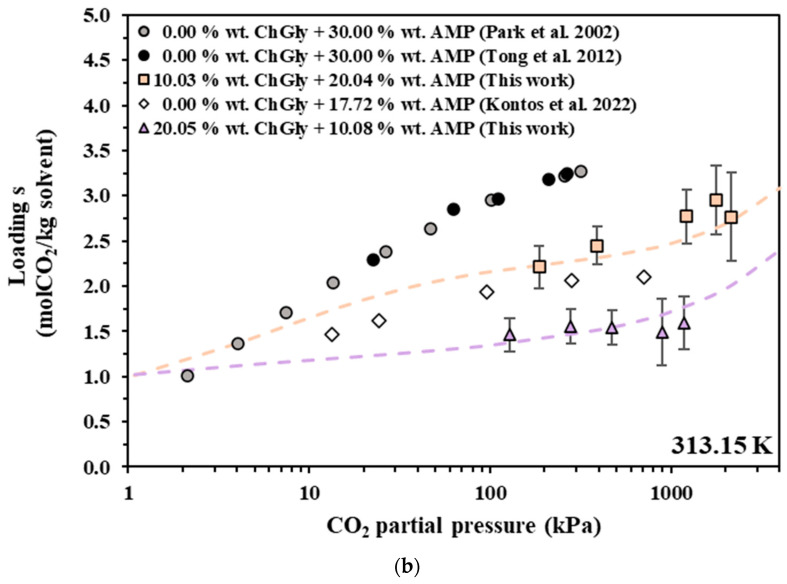
CO_2_ solubility in aqueous $\left[Ch^{+}][Gly^{-}\right]$ + AMP (squares and triangles), AMP 17.72 wt.% (rhombus) [38] and AMP 30.00 wt.% (circles) [50,51] solutions at 313.15 K. Experimental data (squares and triangles) and modified Kent–Eisenberg correlations (dashed lines) for the solubility in $\left[Ch^{+}][Gly^{-}\right]$ + AMP aqueous solutions expressed as (**a**) moles of CO_2_ per mole of amine and (**b**) as moles of CO_2_ per kg of solvent.

In all cases, aqueous solutions of $\left[Bmim^{+}\right]\left[HSO_{4}^{-}\right]$ + AMP, $\left[Bmim^{+}\right]\left[HSO_{4}^{-}\right]$ + MAPA and $\left[Ch^{+}][Gly^{-}\right]$ + AMP, as shown in Table 3, were studied at 298, 313, 323 and 333 K. The new experimental data are shown in Table 4, Table 5, Table 6, Table 7 and Table 8 as moles of CO_2_ per mole of IL + amine. Tables S1–S5 of the Supplementary Material File present the same experimental data as moles of CO_2_ per kg of solvent and as moles of CO_2_ per mole of amine. The uncertainty is estimated via error propagation taking into account the uncertainties of all measurements involved in the solubility calculations (i.e., uncertainties in the weighted masses, the cell volume, the solution density, etc.) and is also presented in Table 4, Table 5, Table 6, Table 7 and Table 8. Finally, some representative results are presented in Figure 2, Figure 3 and Figure 4.

**Table 4:** CO_2_ solubility (*a_d_*, defined as moles of CO_2_ per mole IL + amine) in aqueous $\left[Bmim^{+}][HSO_{4}^{-}\right]$ + AMP (9.93 + 19.94 wt.%) solution.

Temperature ^a^, *T*/K	Total Pressure ^b^, *P*/kPa	$\mathrm{Estimated}\;{\mathrm{CO}}_{2}\;\mathrm{Partial}\;\mathrm{Pressure},\;P_{CO_{2}}/$kPa	${\mathrm{CO}}_{2}\;\mathrm{Loading},\;$ *α_d_*/mol CO_2_ Per mol IL + Amine
298.15	57	54	0.66 ± 0.03
	384	381	0.70 ± 0.03
	819	816	0.73 ± 0.04
	1527	1524	0.78 ± 0.05
313.15	85	79	0.64 ± 0.03
	430	424	0.68 ± 0.03
	896	890	0.70 ± 0.04
	1654	1648	0.75 ± 0.05
323.15	112	101	0.62 ± 0.03
	455	444	0.68 ± 0.03
	934	923	0.69 ± 0.04
	1726	1715	0.75 ± 0.05
333.15	147	129	0.60 ± 0.03
	494	476	0.66 ± 0.03
	979	961	0.69 ± 0.04
	1802	1784	0.74 ± 0.05

^a^ Standard uncertainty in temperature *u*(*T*) = 0.10 K. ^b^ Standard uncertainty in total pressure *u*(*p*) = 0.005∙*P*.

**Table 5:** CO_2_ solubility (*a_d_*, defined as moles of CO_2_ per mole IL + amine) in aqueous $\left[Bmim^{+}][HSO_{4}^{-}\right]$ + AMP (19.74 + 9.92 wt.%) solution.

Temperature ^a^, *T*/K	Total Pressure ^b^, *P*/kPa	$\mathrm{Estimated}\;{\mathrm{CO}}_{2}\;\mathrm{Partial}\;\mathrm{Pressure},\;P_{CO_{2}}/$kPa	${\mathrm{CO}}_{2}\;\mathrm{Loading},\;$ *α_d_*/mol CO_2_ Per mol IL + Amine
298.15	704	701	0.61 ± 0.06
	954	951	0.69 ± 0.06
	1518	1515	0.73 ± 0.07
313.15	757	750	0.59 ± 0.06
	1024	1017	0.67 ± 0.06
	1627	1620	0.72 ± 0.07
323.15	786	775	0.59 ± 0.06
	1073	1061	0.66 ± 0.06
	1703	1692	0.70 ± 0.07
333.15	825	806	0.58 ± 0.06
	1123	1105	0.65 ± 0.06
	1779	1761	0.69 ± 0.07

^a^ Standard uncertainty in temperature *u*(*T*) = 0.10 K. ^b^ Standard uncertainty in total pressure *u*(*p*) = 0.005∙*P*.

**Table 6:** CO_2_ solubility (*a_d_*, defined as moles of CO_2_ per mole IL + amine) in aqueous $\left[Bmim^{+}][HSO_{4}^{-}\right]$ + MAPA (10.01 + 20.00 wt.%) solution.

Temperature ^a^, *T*/K	Total Pressure ^b^, *P*/kPa	$\mathrm{Estimated}\;{\mathrm{CO}}_{2}\;\mathrm{Partial}\;\mathrm{Pressure},\;P_{CO_{2}}/$kPa	${\mathrm{CO}}_{2}\;\mathrm{Loading},\;$ *α_d_*/mol CO_2_ Per mol IL + Amine
313.15	287	281	1.10 ± 0.03
	610	604	1.16 ± 0.04
	849	843	1.31 ± 0.04
323.15	333	322	1.07 ± 0.03
	675	665	1.11 ± 0.04
333.15	364	347	1.05 ± 0.03
	721	704	1.10 ± 0.04

^a^ Standard uncertainty in temperature *u*(*T*) = 0.10 K. ^b^ Standard uncertainty in total pressure *u*(*p*) = 0.005∙*P*.

**Table 7:** CO_2_ solubility (*a_d_*, defined as moles of CO_2_ per mole IL + Amine) in aqueous $\left[Ch^{+}][Gly^{-}\right]$ + AMP (10.03 + 20.04 wt.%) solution.

Temperature, *T*/K ^a^	Total Pressure, *P*/kPa ^b^	$\mathrm{Estimated}\;{\mathrm{CO}}_{2}\;\mathrm{Partial}\;\mathrm{Pressure},\;P_{CO_{2}}/$kPa	${\mathrm{CO}}_{2}\;\mathrm{Loading},\;$ *α_d_*/mol CO_2_ Per mol IL + amine
298.15	175	172	0.80 ± 0.08
	364	362	0.90 ± 0.08
	1152	1150	1.01 ± 0.11
	1677	1675	1.05 ± 0.14
	2016	2014	1.03 ± 0.18
313.15	193	187	0.79 ± 0.08
	398	392	0.87 ± 0.08
	1232	1226	0.99 ± 0.11
	1784	1778	1.05 ± 0.14
	2166	2160	0.98 ± 0.18
323.15	214	203	0.76 ± 0.08
	429	418	0.84 ± 0.08
	1292	1281	0.96 ± 0.11
	1860	1850	1.04 ± 0.14
	2249	2239	1.00 ± 0.18
333.15	239	222	0.73 ± 0.08
	454	437	0.83 ± 0.08
	1345	1328	0.95 ± 0.11
	1943	1926	1.01 ± 0.14
	2349	2331	0.97 ± 0.18

^a^ Standard uncertainty in temperature *u*(*T*) = 0.10 K. ^b^ Standard uncertainty in total pressure *u*(*p*) = 0.005∙*P*.

**Table 8:** CO_2_ solubility (*a_d_*, defined as moles of CO_2_ per mole IL + amine) in aqueous $\left[Ch^{+}][Gly^{-}\right]$ + AMP (20.05 + 10.08 wt.%) solution.

Temperature, *T*/K ^a^	Total Pressure, *P*/kPa ^b^	$\mathrm{Estimated}\;{\mathrm{CO}}_{2}\;\mathrm{Partial}\;\mathrm{Pressure},\;P_{CO_{2}}/$kPa	${\mathrm{CO}}_{2}\;\mathrm{Loading},\;$ *α_d_*/mol CO_2_ Per mol IL + Amine
298.15	113	110	0.68 ± 0.08
	255	252	0.73 ± 0.08
	437	434	0.73 ± 0.08
	831	828	0.75 ± 0.16
	1110	1107	0.74 ± 0.13
313.15	135	129	0.65 ± 0.08
	287	281	0.69 ± 0.09
	481	475	0.69 ± 0.09
	899	893	0.66 ± 0.17
	1186	1180	0.71 ± 0.13
323.15	155	144	0.62 ± 0.08
	313	302	0.66 ± 0.09
	509	498	0.67 ± 0.09
	937	926	0.65 ± 0.17
	1245	1234	0.67 ± 0.13
333.15	179	161	0.59 ± 0.08
	341	323	0.63 ± 0.09
	981	963	0.62 ± 0.17
	1304	1286	0.64 ± 0.13

^a^ Standard uncertainty in temperature *u*(*T*) = 0.10 K. ^b^ Standard uncertainty in total pressure *u*(*p*) = 0.005∙*P*.

In Figure 2, Figure 3 and Figure 4, the experimental data are compared with data from the literature for pure AMP and MAPA aqueous solutions. However, in order to facilitate such comparisons, the obtained experimental CO_2_ solubilities are presented, in Figure 2a, Figure 3a and Figure 4a, as moles of CO_2_ per mole of amine (AMP or MAPA). In more detail, in Figure 2, experimental data of this study that pertain to $\left[Bmim^{+}][HSO_{4}^{-}\right]$ + AMP aqueous solutions are compared with experimental data for the CO_2_ solubility in 17.7 wt.% [38] and 30.00 wt.% [50,51] AMP aqueous solution, while in Figure 3, the experimental data for the $\left[Bmim^{+}][HSO_{4}^{-}\right]$ + MAPA (10.01 + 20.00 wt.%) aqueous solution are compared with the data from the literature for the 17.88 wt.% [52] and 30.06 wt.% [38] MAPA aqueous solution. Finally, in Figure 4, solubility data of $\left[Ch^{+}][Gly^{-}\right]$ + AMP are compared with experimental data for the CO_2_ solubility in 17.7 wt.% [38] and 30.00 wt.% [50,51] AMP aqueous solution.

#### 4.1.1. Replacing Amine with $\left[Bmim^{+}][HSO_{4}^{-}\right]$

The $\left[Bmim^{+}][HSO_{4}^{-}\right]$-based solutions under investigation can be regarded modified neat amine solutions, in which a portion of the amine has been substituted with $\left[Bmim^{+}][HSO_{4}^{-}\right]$. Consequently, the aqueous $\left[Bmim^{+}][HSO_{4}^{-}\right]$ solution containing 19.74 wt.% AMP or 20.00 wt.% MAPA can be obtained from a 30 wt.% neat amine solution by replacing almost 10 wt.% of the amine by an equal mass of $\left[Bmim^{+}][HSO_{4}^{-}\right]$.

In general, CO_2_ solubility, in terms of CO_2_ per mole of amine, increases with decreasing amine concentration in the initial aqueous solution. This general observation is illustrated, for example, in Figure 3a, where the loading of 17.88 wt.% and 30.06 wt.% MAPA aqueous solutions are compared. It is clear that the loading (in moles of CO_2_ per mole of amine) is higher in the less concentrated amine solution, i.e., 17.88 wt.% in this case. However, $\left[Bmim^{+}][HSO_{4}^{-}\right]$-based solutions show CO_2_ solubility lower than that of the neat amine solutions. In more detail, the CO_2_ loading in the aqueous $\left[Bmim^{+}][HSO_{4}^{-}\right]$ solution containing 9.93 wt.% AMP is similar to that of the 17.72 wt.% AMP solution, although it was expected to be higher (Figure 2a). Similarly, the CO_2_ solubility of the aqueous $\left[Bmim^{+}][HSO_{4}^{-}\right]$-AMP solution containing 19.94 wt.% AMP is lower than that of the 30.00 wt.% neat AMP solution, although it was expected to fall within the solubility of the 30.00 and 17.72% neat AMP solutions (Figure 2a). Similar behavior occurs in aqueous $\left[Bmim^{+}][HSO_{4}^{-}\right]$ solution containing 20.00 wt.% MAPA, where the CO_2_ solubility is similar to that of the 30.06 wt.% neat MAPA solution, although it was expected to fall between the solubility of 30.06 and 17.88 wt.% neat MAPA solutions (Figure 3a). Therefore, replacing part of the amine with an equal mass of $\left[Bmim^{+}][HSO_{4}^{-}\right]$ results in decreased CO_2_ solubility, in terms of CO_2_ uptake per mole of amine.

Two primary factors should be considered to explain these observations: the influence of $\left[Bmim^{+}][HSO_{4}^{-}\right]$ on the solution’s basicity and the introduction of new sites for physical interactions with CO_2_ resulting from the addition of the salt. $\left[Bmim^{+}\right]$ has no acid or acidic groups; therefore, it will not react with bases. The slightly acidic character of the $\left[Bmim^{+}][HSO_{4}^{-}\right]$ is mainly attributed to the acidic hydrogen of the bisulfate, ${HSO}_{4}^{-}$, anion. Since, in alkaline solutions, ${HSO}_{4}^{-}$ reacts with OH^−^, forming the sulfate anion ${SO}_{4}^{-2}$ and water. This reaction tends to lower the solution’s basicity, therby reducing its capacity for chemical absorption of the acidic gas (CO_2_). In other words, the introduction of ${HSO}_{4}^{-}$ increases the $\left[H^{+}\right]$ concentration and shifts the reaction (8) towards the formation of more ${R_{a}R_{b}NH}_{2}^{+}$, thus reducing the available amine for CO_2_ absorption. However, the well-known strong intermolecular interactions of the CO_2_ molecule with the S=O group [33] tend to increase the CO_2_ physical absorption. Thus, the presence of sulfate anions, ${SO}_{4}^{-2}$, introduces additional sites that facilitate favorable intramolecular interactions with CO_2_. Collectively, these factors contribute to the observed outcome: the substitution of amine with $\left[Bmim^{+}][HSO_{4}^{-}\right]$ decreases the CO_2_ solubility, in terms of CO_2_ per mole of amine.

Nevertheless, in industrial applications, solvent performance is typically assesed by comparing the CO_2_ solubility in terms of moles of CO_2_ per kg of solvent. In general, solutions with higher concentrations have higher CO_2_ loading, in terms of moles of CO_2_ per kg of solvent, than diluted solutions under the same conditions. Keeping this in mind, the CO_2_ solubility of the $\left[Bmim^{+}][HSO_{4}^{-}\right]$-based solutions are expected to be similar to that of the neat amine solutions with the same amine content. However, the CO_2_ solubility of the aqueous $\left[Bmim^{+}][HSO_{4}^{-}\right]$-AMP solution containing 19.94 wt.% AMP is lower than that of the 17.72% neat AMP solution (Figure 2b), while that of the aqueous $\left[Bmim^{+}][HSO_{4}^{-}\right]$ solution containing 20.00 wt.% MAPA is similar to that of 17.88 wt.% neat MAPA solution (Figure 3a).

#### 4.1.2. Replacing Amine with $\left[Ch^{+}][Gly^{-}\right]$

Similarly to the thoughts presented at the beginning of the previous section, $\left[Ch^{+}][Gly^{-}\right]$-AMP solutions can be regarded modified neat AMP solutions by replacing a portion of AMP with $\left[Ch^{+}][Gly^{-}\right]$. Consequently, the 10.03 wt.% $\left[Ch^{+}][Gly^{-}\right]$ + 20.04 wt.% AMP aqueous solution can be obtained from a 30 wt.% neat AMP solution by replacing almost 10 wt.% of AMP with an equal mass of $\left[Ch^{+}][Gly^{-}\right]$. However, in contrast to the case described in the previous section, the CO_2_ solubility of the aqueous $\left[Ch^{+}][Gly^{-}\right]$-AMP solution containing 20.04 wt.% AMP is slightly higher than that of the 17.72 wt.% neat AMP solution, although a lower solubility was expected (Figure 4a). As mentioned above, CO_2_ solubility, in terms of CO_2_ per mole of amine, typically increases with decreasing amine concentration. Consequently, it can be concluded that replacing an amount of AMP with an equal mass of $\left[Ch^{+}][Gly^{-}\right]$ increases the CO_2_ solubility, in terms of CO_2_ per mole of amine.

**Figure 5:**
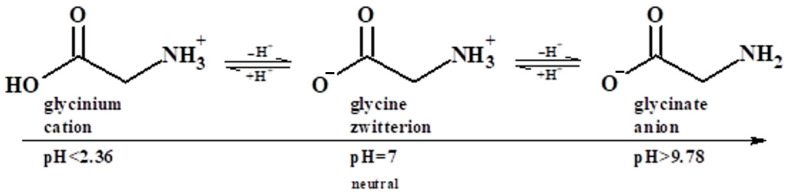
Ionic forms of glycine as a function of the acidity of the solution.

As in the case of $\left[Bmim^{+}][HSO_{4}^{-}\right]$-based solutions, two main factors should be taken into consideration to explain such observations: the effect of the $\left[Ch^{+}][Gly^{-}\right]$ salt on the basicity of the solution and the new sites for physical and chemical interactions with CO_2_ that are introduced upon the addition of the salt. It is well known that the acidic or basic character of glycine zwitterion depends on the pH of the solution in which glycine is introduced. In more detail, in acidic solutions, with a pH lower than 2.36, glycine acts as a base and its amine group is protonated, forming the glycinate cation (Figure 5), while in basic solutions, with a pH higher than 9.78, glycine acts as an acid and the carboxylate group is deprotonated, resulting in the glycinium anion (Figure 5) [53,54]. Thus, Gly^−^ anion, in the alkaline environment of the amine, introduces available amine groups for favorable chemical absorption of CO_2_, and, consequently, contributes to the total CO_2_ absorption. In other words, since Gly^−^ anion acts as a weak base, due to the presence of amine groups, it tends to increase the basicity of the solution. However, the choline cation, Ch^+^, acts as a weak acid, tending to decrease the basicity of the solution and, consequently, the chemical absorption of the acid gas (CO_2_). Finally, the addition of the $\left[Ch^{+}][Gly^{-}\right]$ introduces new available sites for intermolecular interactions with CO_2_, such as the alcoholic–OH group in Ch^+^, which tends to increase the physical CO_2_ solubility [40]. The net effect of all these factors is described by the observation mentioned above, i.e., the replacement of amine with $\left[Ch^{+}][Gly^{-}\right]$ increases the CO_2_ solubility, in terms of CO_2_ per mole of amine.

As mentioned above, in industrial practice, the evaluation of solvent performance is usually based on comparisons of solubility expressed as moles of CO_2_ per kg of solvent. In general, solutions with higher concentrations present higher CO_2_ loading, in terms of moles of CO_2_ per kg of solvent, than diluted solutions under the same conditions. Keeping this in mind, the CO_2_ solubility of the $\;[Ch^{+}][Gly^{-}]$-based solutions are expected to be similar to those of the neat amine solutions. Actually, the CO_2_ solubility of the aqueous $\left[Ch^{+}][Gly^{-}\right]$-AMP solution containing 20.04 wt.% AMP falls within the solubility of 17.72 and 30.00 wt.% neat AMP solutions (Figure 4b); thus, the replacement of amine with $\left[Ch^{+}][Gly^{-}\right]$ decreases the CO_2_ solubility, in terms of moles of CO_2_ per kg of solvent.

#### 4.1.3. Replacing Water with $\left[Ch^{+}][Gly^{-}\right]$

Interestingly, such aqueous IL–amine solutions can be evaluated from a different perspective: they may be considered as systems derived from neat amine aqueous solutions by replacing a portion of water with $\left[Ch^{+}][Gly^{-}\right]$. In other words, the 10.03 wt.% $\left[Ch^{+}][Gly^{-}\right]$ + 20.04 wt.% AMP aqueous solution can be obtained from a 20.04 wt.% neat AMP solution by removing 9.96 wt.% of water and replacing it with an equal mass of $\left[Ch^{+}][Gly^{-}\right]$. Bearing this in mind, the results of Figure 4b reveal that replacing water with $\left[Ch^{+}][Gly^{-}\right]$ does not significantly affect the CO_2_ solubility in the bulk (expressed as moles of CO_2_ per kg of solvent). That is, the CO_2_ solubility in the aqueous $\left[Ch^{+}][Gly^{-}\right]$-AMP solution containing 20.04 wt.% AMP is comparable to that of the 20.04 wt.% neat AMP. These results suggest the feasibility of replacing part of the water content with $\left[Ch^{+}][Gly^{-}\right]$ without substantially altering bulk CO_2_ absorption characteristics (solubility expressed as moles of CO_2_ per kg of solvent). Moreover, this substitution is expected to lower the solvent’s vapor pressure, and, given that $\left[Ch^{+}][Gly^{-}\right]$ is non-toxic, the resulting solvent is unlikely to pose increased environmental hazards, although it is more expensive.

### 4.2. Modeling Results

Carbamates are formed upon reaction of CO_2_ with primary and secondary amines in the presence of water. Based on this reaction mechanism, the stoichiometric limit of CO_2_ chemical absorption is 0.5 moles of CO_2_ per mole of amine. This limit may be exceeded, mainly at high pressures, due to significant CO_2_ molecular dissolution (physical absorption) and carbamate hydrolysis. However, the produced carbamate is unstable and easily hydrolyzed in cases of sterically hindered primary and secondary amines.

The reaction mechanism should be a priori assumed to apply the Kent–Eisenberg model. In this direction, in the current study, MAPA was modeled as a diamine that consists of one primary and one secondary amine group, using the approach described in detail by Kontos et al. (2022) [38]. In this contex, MAPA was modeled under the assumption of equal and independent reactivity for each amine group, i.e., each functional group reacts independently, with its reactivity unaffected by the potential reaction of the other one. This approach is similar to Flory’s principle of independent reactivity, which is commonly applied in the modeling of polymerization reactions [38,55]. This approach was successfully applied by Kontos et al. to model, using the modified Kent–Eisenberg model, the CO_2_ absorption by aqueous solutions of pure MAPA [38] and deep eutectic solvents containing MAPA [55]. Also, AMP was modeled as a non-carbamate-forming amine, since it presents sterical hindrance, an approach often applied in the literature [38,44].

Considering the $\left[Bmim^{+}\right]\left[HSO_{4}^{-}\right]$-containing mixtures, i.e., $\left[Bmim^{+}\right]\left[HSO_{4}^{-}\right]$ + MAPA and $\left[Bmim^{+}\right]\left[HSO_{4}^{-}\right]$ + AMP, it was assumed that only the amine (AMP or MAPA) is capable of chemical absorption of CO_2_. In this way, the Kent–Eisenberg equations that refer to a single-carbamate or non-carbamate-forming amines were used for systems with MAPA or AMP, respectively. Furthermore, parameters reported in the literature were applied to estimate $K_{1}$ through $K_{5}$ using Equation (34). The MAPA and AMP parameters corresponding to $K_{1}$ as well as the MAPA parameters for $K_{5}$ were adopted from Kontos et al. [38], while parameters adopted from Edwards et al. [56] were used for $K_{2}$ through $K_{4}$, as presented in Table 9. For the estimation of Henry’s constant in aqueous amine solutions, usually the parameters of Edwards et al. [56] are used, which refer to pure water. Bearing in mind that, except for the amine, the investigated solutions of this study also contain ILs, which are expected to affect the physical absorption of CO_2_, new parameters of Equation (18) for Henry’s constants were adjusted to the experimental data. Table 9 provides a summary of all parameters used and their corresponding sources.

Considering the $\left[Ch^{+}][Gly^{-}\right]$ + AMP mixtures, it was assumed that both the $\left[Gly^{-}\right]$ and AMP amino groups are capable of chemical absorption of CO_2_. Thus, the modeling problem refers to a mixture of one carbamate-forming amine, i.e., the $\left[Gly^{-}\right]$ anion, and one non-carbamate-forming amine, i.e., the AMP. Similar to the previous system, the AMP parameters for the estimation of $K_{1}$ were adopted from Kontos et al., while the parameters for $K_{2}$ through $K_{4}$ were adopted from Edwards et al., as presented in Table 9. Furthermore, the needed glycine parameters for $K_{1}$ and $K_{5}$ were adjusted to the experimental data of this study. However, to keep the number of adjusted parameters low, only the *A* and *D* parameters of Equation (34) were estimated, while the parameters of Edwards et al. [56] were used for the approximation of Henry’s constant, as presented in Table 9.

Using the parameters of Table 9, the model correlations present a satisfactory agreement with the experimental data, as shown in Figure 2, Figure 3 and Figure 4. As presented in Table 10, deviations ranging between 2.0 and 11.6% were obtained in all cases.

**Table 9:** Parameters A to D of equilibrium constant *K* (Equation (34)) for the studied aqueous solutions.

Parameter	Units	A	B	C	D	Regression Range (K)	Reference
$K_{1AMP}$	mol/kg	−5936.63	0	0	−3.1347	313–383	[38]
$K_{1\;MAPA}$	mol/kg	−6164.85	0	0	−4.1080	313–383	[38]
$K_{5\;MAPA}$	mol/kg	−3534.70	0	0	7.4398	313–383	[38]
$K_{1\;Glycine}$	mol/kg	−5175.64	0	0	−3.8531	298–333	This work
$K_{5\;Glycine}$	mol/kg	−8815.32	0	0	23.6100	298–333	This work
$K_{2}$	mol/kg	−12,092.10	−36.7816	0	235.482	273–498	[56]
$K_{3}$	mol/kg	−12,431.70	−35.4819	0	220.067	273–498	[56]
$K_{4}$	${mol}^{2}/{kg}^{2}$	−13,445.90	−22.4773	0	140.932	273–498	[56]
$H_{{CO}_{2}}$ ([Bmim^+^][HSO_4_^−^] + AMP, 9.93 + 19.94 wt.%)	atm kg/mol	−628.00	0	0	7.600	298–333	This work
$H_{{CO}_{2}}$ ([Bmim^+^][HSO_4_^−^] + AMP, 19.74 + 9.92 wt.%)	atm kg/mol	−1037.00	0	0	7.000	298–333	This work
$H_{{CO}_{2}}$ ([Bmim^+^][HSO_4_^−^] + MAPA)	atm kg/mol	−1785.86	0	0	10.33	313–333	This work
$H_{{CO}_{2}}$ [Ch^+^][Gly^−^] + AMP	atm kg/mol	−6789.04	−11.4519	−0.010454	94.4914	273–498	[56]

**Table 10:** Percentage average absolute deviations (%AAD) of model’s predictions from the experimental data of this study.

System	%AAD ^a^
$\left[Bmim^{+}\right]\left[HSO_{4}^{-}\right]$+ AMP, 9.93 + 19.94 wt.%	10.0
$\left[Bmim^{+}\right]\left[HSO_{4}^{-}\right]$+ AMP, 19.74 + 9.92 wt.%	2.7
$\left[Bmim^{+}\right]\left[HSO_{4}^{-}\right]$+ MAPA, 10.01 + 20.00 wt.%	2.0
$\left[Ch^{+}][Gly^{-}\right]$+ AMP, 10.03 + 20.04 wt.%	5.5
$\left[Ch^{+}][Gly^{-}\right]$+ AMP, 20.04 + 10.08 wt.%	11.6

^a^ %AAD=100ndata∑aexp−acalcaexp, where $a_{exp}$ and $a_{calc}$ stand for the experimental and calculated CO_2_ loading (expressed as moles of CO_2_ per mole of amine), respectively, and *n_data_* is the total number of data.

**Figure 6:**
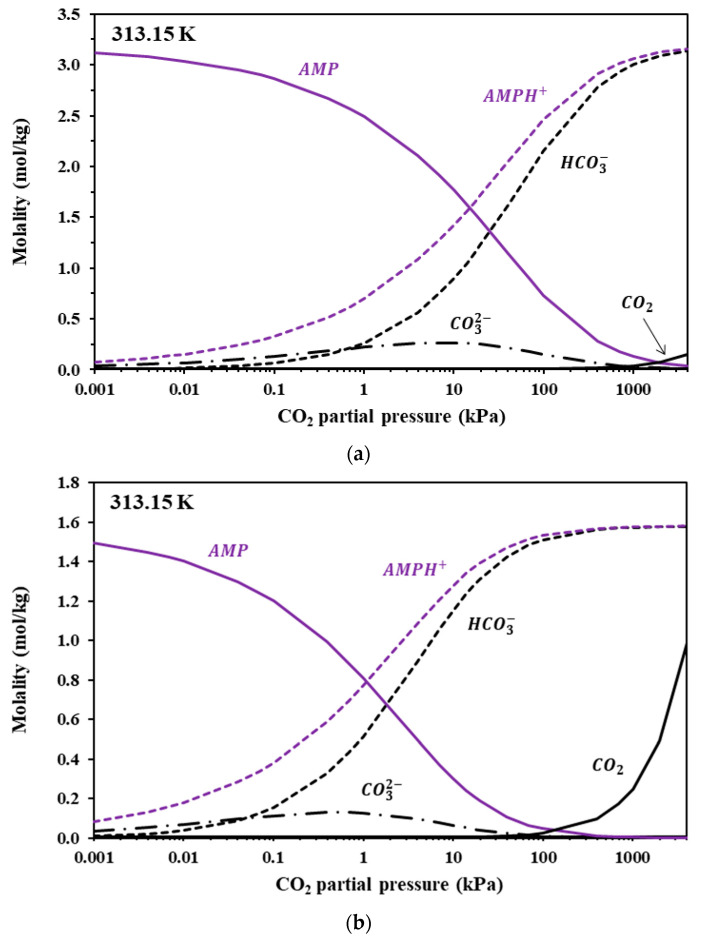
Liquid-phase speciation in CO_2_-loaded aqueous solutions of (**a**) $\left[Bmim^{+}\right]\left[HSO_{4}^{-}\right]$ + AMP (9.93 + 19.94% wt) and (**b**) $\left[Bmim^{+}\right]\left[HSO_{4}^{-}\right]$ + AMP (19.74 + 9.92 wt.%) at 313.15 K. Compositions were calculated using the modified Kent–Eisenberg model (presented in this work).

**Figure 7:**
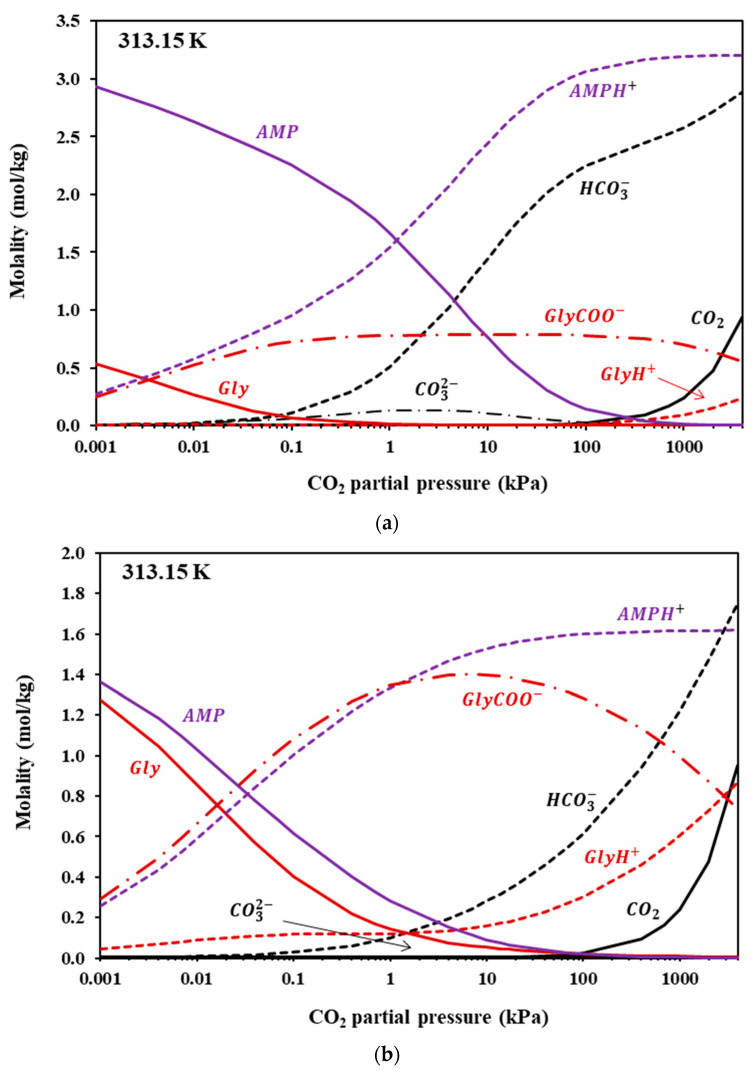
Liquid-phase speciation in CO_2_-loaded aqueous solutions of (**a**) $\left[Ch^{+}][Gly^{-}\right]$ + AMP (10.03 + 20.04 wt.%) and (**b**) $\left[Ch^{+}][Gly^{-}\right]$ + AMP (20.05 + 10.08 wt.%) at 313.15 K. Compositions were calculated using the modified Kent–Eisenberg model (presented in this work).

**Figure 8:**
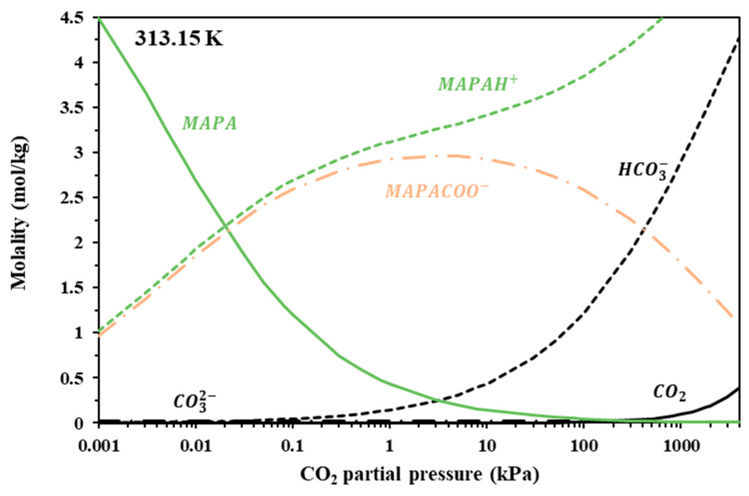
Liquid-phase speciation in CO_2_-loaded aqueous solutions of $\left[Bmim^{+}\right]\left[HSO_{4}^{-}\right]$ + MAPA (10.01 + 20.00% wt) at 313.15 K. Compositions were calculated using the modified Kent–Eisenberg model (presented in this work).

Since the modified K-E model successfully represents experimental CO_2_ solubility data, it could be applied to predict the liquid speciation in the CO_2_-loaded IL + Amine aqueous solutions. Figure 6, Figure 7 and Figure 8 illustrate the liquid speciation predicted by the model for the investigated IL + Am aqueous solutions at 313 K. The comparison of such figures reveals that the free amine concentration of the non-sterically hindered amine groups, i.e., in [Gly^−^] and in MAPA, becomes low at very low CO_2_ partial pressures. On the contrary, the free amine concentration of the sterically hindered AMP presents a more moderate reduction. This is clearly shown in Table 11, in which the free amine content at 313 K and 10 kPa of CO_2_ partial pressure is presented, as predicted by the model, for all investigated solutions. Also, as shown in Figure 7 and Figure 8, the carbamate concentration presents a maximum at approximately 1–10 kPa, for both [Gly^−^] and MAPA. Beyond this range, the increase in CO_2_ solubility is primarily attributed to carbamate hydrolysis and to physical dissolution of molecular CO_2_.

**Table 11:** Free amine content of all investigated solutions at 313 K and 10 kPa of CO_2_ partial pressure.

System	Free Amine (% of the Initial)		
	AMP	MAPA	$\left[Gly^{-}\right]$
$\left[Bmim^{+}\right]\left[HSO_{4}^{-}\right]$ + AMP, 9.93 + 19.94 wt.%	55.3	-	-
$\left[Bmim^{+}\right]\left[HSO_{4}^{-}\right]$ + AMP, 19.74 + 9.92 wt.%	19.1	-	-
$\left[Bmim^{+}\right]\left[HSO_{4}^{-}\right]$ + MAPA, 10.01 + 20.00 wt.%	-	2.2	-
$\left[Ch^{+}][Gly^{-}\right]$ + AMP, 10.03 + 20.04% wt	23.6	-	0.7
$\left[Ch^{+}][Gly^{-}\right]$ + AMP, 20.04 + 10.08 wt.%	5.7	-	3.3

## 5. Conclusions

The solubility of CO_2_ in aqueous solutions of $\left[Bmim^{+}\right]\left[HSO_{4}^{-}\right]$ + AMP, $\left[Bmim^{+}\right]\left[HSO_{4}^{-}\right]$ + MAPA and $\left[Ch^{+}][Gly^{-}\right]$ + AMP was experimentally investigated. For $\left[Bmim^{+}\right]\left[HSO_{4}^{-}\right]$-based solutions, it was shown that starting from an aqueous amine solution, replacing a small portion of the amine with $\left[Bmim^{+}\right]\left[HSO_{4}^{-}\right]$ significantly decreases CO_2_ solubility, expressed as moles of CO_2_ per mole of amine, since such replacement lowers the solution’s basicity, reducing the chemical absorption of the acidic gas (CO_2_), despite introducing new sites for physical interactions.

In contrast, for $\left[Ch^{+}][Gly^{-}\right]$-based solutions, replacing a small part of the amine with $\left[Ch^{+}][Gly^{-}\right]$ increases the CO_2_ solubility, expressed as moles of CO_2_ per mole of amine, since new sites for physical and chemical interactions with CO_2_ are introduced.

Finally, it was shown that, starting from an aqueous amine solution, if a small part of water is replaced by $\left[Ch^{+}][Gly^{-}\right]$, the CO_2_ solubility in the bulk (expressed as moles of CO_2_ per kg of solvent) is not significantly altered. However, such replacement is expected to lower the vapor pressure of the solvent and, since $\left[Ch^{+}][Gly^{-}\right]$ is non-toxic, the new solvent is not expected to be more environmentally hazardous, although more expensive.

To predict the CO_2_ solubility in the investigated IL-amine solutions, the modified Kent–Eisenberg model was applied. The model predictions show that the free AMP content (amine that remained unreacted) becomes very low at CO_2_ partial pressures above approximately 100–200 kPa. In contrast, the free amine content of the non-sterically hindered amine groups, i.e., in $\left[Gly^{-}\right]$, and MAPA, is very low already from relatively modest CO_2_ partial pressures, on the order of 1–10 kPa. Consequently, the increase in CO_2_ solubility at higher partial pressures is mainly attributed to carbamate hydrolysis, along with a non-negligible contribution from molecular CO_2_ dissolution.

## Data Availability

All data is included in the article.

## Supplementary Materials

The following supporting information can be downloaded at: https://www.mdpi.com/article/10.3390/molecules30183832/s1, Table S1: CO_2_ solubility in aqueous $\left[Bmim^{+}][HSO_{4}^{-}\right]$ + AMP (9.93 + 19.94 wt.%) solution; Table S2: CO_2_ solubility in aqueous $\left[Bmim^{+}][HSO_{4}^{-}\right]$ + AMP (19.74 + 9.92 wt.%) solution; Table S3: CO_2_ solubility in aqueous $\left[Bmim^{+}][HSO_{4}^{-}\right]$ + MAPA (10.01 + 20.00 wt.%) solution; Table S4: CO_2_ solubility in aqueous $\left[Ch^{+}][Gly^{-}\right]$ + AMP (10.03 + 20.04 wt.%) solution; Table S5: CO_2_ solubility in aqueous $\left[Ch^{+}][Gly^{-}\right]$ + AMP (20.05 + 10.08 wt.%) solution.

## Abbreviations

The following abbreviations and symbols are used in this manuscript:

AMP	2-Amino-2-methyl-1-propanol
${\left[Bf_{4}\right]}^{-}$	Tetrafluoroborate anion
BASIL	Biphasic acid scavenging utilizing ionic liquids
$\left[Bmim^{+}\right]\left[HSO_{4}^{-}\right]$	1-Butyl-3-methylimidazolium hydrogen sulfate
$\left[Bmim^{+}\right]\left[Ac^{-}\right]$	1-Butyl-3-methylimidazolium acetate
${d_{CO}}_{2}$	CO_2_ density
$d_{sol}$	Solvent density
CCS	Carbon capture and storage
$\left[Ch^{+}][Gly^{-}\right]$	Choline glycine
$\left[Emim^{+}\right]\left[HSO_{4}^{-}\right]$	1-Ethyl-3-methylimidazolium hydrogen sulfate
$H_{CO2}$	Henry’s constant
$\left[Hmim^{+}\right]\left[Gly^{-}\right]$	1-Hexyl-3-methylimidazolium glycinate
ILs	Ionic liquids
MAPA	3-(Methylamino)propylamine
$m_{CO_{2}}^{L}$	Mass of CO_2_ absorbed in the liquid phase
$m_{sol}$	Mass of the solvent
MDEA	*N*-methyl-diethanolamine
*n*	Total moles of CO_2_ loaded in the equilibrium cell
$n_{A}$	Moles of component *A*
$n_{CO_{2}}^{L}$	Moles of CO_2_ absorbed in the liquid phase
NG	Natural gas
$P_{CO2}$	Vapor phase CO_2_ partial pressure
${\left[Pf_{6}\right]}^{-}$	Hexafluorophosphate anion
$R_{a}R_{b}NH$	Carbamate-forming amine
${\left[Tf_{2}N\right]}^{-}$	Bis(trifluoromethylsulfonyl)imide anion
${\left[TfA\right]}^{-}$	Trifluoroacetate anion
${\left[TfO\right]}^{-}$	Trifluoromethanesulfonate anion
$V_{T}$	Volume of the cell
$V_{sol}$	Volume of the solvent
VOCs	Volatile organic compounds
