# Supplementary material for: CO2 Solubility in Aqueous Solutions of Amine–Ionic Liquid Blends: Experimental Data for Mixtures with AMP and MAPA and Modeling with the Modified Kent–Eisenberg Model
Source: Molecules. 2025 Sep 21;30(18):3832. doi: 10.3390/molecules30183832 (PMC12472619; doi:10.3390/molecules30183832)
Supplement: Supplementary file 1 [file molecules-30-03832-s001.zip › molecules-3824744-supplementary.pdf]

Supplementary Material for

CO_2 Solubility in Aqueous Solutions of Amine–Ionic Liquid Blends: Experimental Data for Mixtures with AMP and MAPA and Modeling with the Modified Kent–Eisenberg Model

Giannis Kontos and Ioannis Tsivintzelis *

Department of Chemical Engineering, Aristotle University of Thessaloniki, 54124 Thessaloniki, Greece

* Correspondence: tioannis@cheng.auth.gr

Table S1. CO_2 solubility of in aqueous $[Bmim^+][HSO_4^-]$+ AMP (9.93 + 19.94 %wt.) solution.

Temperature[a], T / K	Total Pressure[b], P / kPa	Estimated CO_2 partial pressure, P_{CO_2}/ kPa	CO_2 loading, α_d / mol CO_2 per mol IL+amine	CO_2 loading, a_a / mol CO_2 per mol AMP	CO_2 loading, s / mol CO_2 per kg solvent
298.2	57	54	0.66 ± 0.03	0.78 ± 0.03	1.76 ± 0.08
	384	381	0.70 ± 0.03	0.84 ± 0.04	1.87 ± 0.08
	819	816	0.73 ± 0.04	0.86 ± 0.05	1.93 ± 0.11
	1527	1524	0.78 ± 0.05	0.93 ± 0.06	2.08 ± 0.13
313.2	85	79	0.64 ± 0.03	0.76 ± 0.04	1.71 ± 0.08
	430	424	0.68 ± 0.03	0.81 ± 0.04	1.82 ± 0.08
	896	890	0.70 ± 0.04	0.83 ± 0.05	1.85 ± 0.11
	1654	1648	0.75 ± 0.05	0.89 ± 0.06	2.00 ± 0.13
323.2	112	101	0.62 ± 0.03	0.74 ± 0.04	1.66 ± 0.08
	455	444	0.68 ± 0.03	0.81 ± 0.04	1.80 ± 0.08
	934	923	0.69 ± 0.04	0.82 ± 0.05	1.84 ± 0.11
	1726	1715	0.75 ± 0.05	0.89 ± 0.06	1.98 ± 0.13
333.2	147	129	0.60 ± 0.03	0.72 ± 0.04	1.60 ± 0.08
	494	476	0.66 ± 0.03	0.79 ± 0.04	1.76 ± 0.08
	979	961	0.69 ± 0.04	0.82 ± 0.05	1.83 ± 0.11
	1802	1784	0.74 ± 0.05	0.88 ± 0.06	1.97 ± 0.13

[a]Standard uncertainty in temperature u(T) = 0.10 K

[b]Standard uncertainty in total pressure u(p) = 0.005·P

Table S2. CO_2 solubility in aqueous $[Bmim^+][HSO_4^-]$ + AMP (19.74 + 9.92 %wt.) solution.

Temperature[a], T / K	Total Pressure[b], P / kPa	Estimated CO_2 partial pressure, P_{CO_2}/ kPa	CO_2 loading, α_d / mol CO_2 per mol IL+amine	CO_2 loading, a_a / mol CO_2 per mol AMP	CO_2 loading, s / mol CO_2 per kg solvent
298.2	704	701	0.61 ± 0.06	1.06 ± 0.10	1.18 ± 0.11
	954	951	0.69 ± 0.06	1.20 ± 0.10	1.34 ± 0.12
	1518	1515	0.73 ± 0.07	1.28 ± 0.12	1.43 ± 0.13
313.2	757	750	0.59 ± 0.06	1.03 ± 0.10	1.14 ± 0.11
	1024	1017	0.67 ± 0.06	1.17 ± 0.10	1.30 ± 0.12
	1627	1620	0.72 ± 0.07	1.25 ± 0.12	1.39 ± 0.13
323.2	786	775	0.59 ± 0.06	1.03 ± 0.10	1.15 ± 0.11
	1073	1061	0.66 ± 0.06	1.15 ± 0.11	1.28 ± 0.12
	1703	1692	0.70 ± 0.07	1.23 ± 0.12	1.37 ± 0.13
333.2	825	806	0.58 ± 0.06	1.02 ± 0.10	1.13 ± 0.11
	1123	1105	0.65 ± 0.06	1.13 ± 0.11	1.26 ± 0.12
	1779	1761	0.69 ± 0.07	1.21 ± 0.12	1.34 ± 0.13

[a]Standard uncertainty in temperature u(T) = 0.10 K

[b]Standard uncertainty in total pressure u(p) = 0.005·P

Table S3. CO_2 solubility in aqueous $[Bmim^+][HSO_4^-]$+ MAPA (10.01 + 20.00 %wt.) solution.

Temperature[a], T / K	Total Pressure[b], P / kPa	Estimated CO_2 partial pressure, P_{CO_2}/ kPa	CO_2 loading, α_d / mol CO_2 per mol IL+amine	CO_2 loading, a_a / mol CO_2 per mol MAPA	CO_2 loading, s / mol CO_2 per kg solvent
313.2	287	281	1.10 ± 0.03	1.30 ± 0.04	2.95 ± 0.09
	610	604	1.16 ± 0.04	1.37 ± 0.05	3.11 ± 0.10
	849	843	1.31 ± 0.04	1.55 ± 0.05	3.53 ± 0.10
323.2	333	322	1.07 ± 0.03	1.26 ± 0.04	2.87 ± 0.09
	675	664	1.11 ± 0.04	1.32 ± 0.05	3.00 ± 0.10
333.2	364	347	1.05 ± 0.03	1.25 ± 0.04	2.83 ± 0.09
	721	704	1.10 ± 0.04	1.30 ± 0.05	2.95 ± 0.11

[a]Standard uncertainty in temperature u(T) = 0.10 K

[b]Standard uncertainty in total pressure u(p) = 0.005·P

Table S4. CO_2 solubility in aqueous $[Ch^+][Gly^-]$ + AMP (10.03 + 20.04 %wt.) solution.

**Temperature**[a], ***T*** **/ K**	**Total Pressure**[b], ***P*** **/ kPa**	**Estimated CO_2 partial pressure, P_{CO_2}/ kPa**	**CO_2 loading, α_d / mol CO_2 per mol IL+amine**	**CO_2 loading, a_a / mol CO_2 per mol AMP**	**CO_2 loading, *s* / mol CO_2 per kg solvent**
298.2	175	172	0.80 ± 0.08	1.00 ± 0.10	2.25 ± 0.23
	364	362	0.90 ± 0.08	1.12 ± 0.10	2.53 ± 0.21
	1152	1150	1.01 ± 0.11	1.27 ± 0.13	2.85 ± 0.30
	1677	1675	1.05 ± 0.14	1.31 ± 0.17	2.94 ± 0.38
	2016	2014	1.03 ± 0.18	1.29 ± 0.22	2.90 ± 0.49
313.2	193	187	0.79 ± 0.08	0.98 ± 0.10	2.21 ± 0.23
	398	392	0.87 ± 0.08	1.09 ± 0.10	2.45 ± 0.21
	1232	1226	0.99 ± 0.11	1.23 ± 0.14	2.77 ± 0.30
	1784	1778	1.05 ± 0.14	1.32 ± 0.17	2.96 ± 0.38
	2166	2160	0.98 ± 0.18	1.23 ± 0.22	2.77 ± 0.49
323.2	214	203	0.76 ± 0.08	0.95 ± 0.11	2.14 ± 0.23
	429	418	0.84 ± 0.08	1.05 ± 0.10	2.37 ± 0.21
	1292	1281	0.96 ± 0.11	1.20 ± 0.14	2.69 ± 0.30
	1860	1850	1.04 ± 0.14	1.30 ± 0.17	2.92 ± 0.38
	2249	2239	1.00 ± 0.18	1.26 ± 0.22	2.82 ± 0.49
333.2	239	222	0.73 ± 0.08	0.92 ± 0.11	2.06 ± 0.23
	454	437	0.83 ± 0.08	1.04 ± 0.10	2.34 ± 0.21
	1345	1328	0.95 ± 0.11	1.19 ± 0.14	2.67 ± 0.30
	1943	1926	1.01 ± 0.14	1.27 ± 0.17	2.84 ± 0.38
	2349	2331	0.97 ± 0.18	1.22 ± 0.22	2.73 ± 0.49

[a]Standard uncertainty in temperature u(T) = 0.10 K

[b]Standard uncertainty in total pressure u(p) = 0.005·P

Table S5. CO_2 solubility in aqueous $[Ch^+][Gly^-]$ + AMP (20.05 + 10.08 %wt.) solution.

Temperature[a], T / K	Total Pressure[b], P / kPa	Estimated CO_2 partial pressure, P_{CO_2}/ kPa	CO_2 loading, α_d / mol CO_2 per mol IL+amine	CO_2 loading, a_a / mol CO_2 per mol AMP	CO_2 loading, s / mol CO_2 per kg solvent
298.2	113	110	0.68 ± 0.08	1.36 ± 0.16	1.53 ± 0.18
	255	252	0.73 ± 0.08	1.45 ± 0.17	1.64 ± 0.19
	437	434	0.73 ± 0.08	1.46 ± 0.16	1.64 ± 0.19
	831	828	0.75 ± 0.16	1.51 ± 0.33	1.70 ± 0.37
	1110	1107	0.74 ± 0.13	1.48 ± 0.26	1.67 ± 0.29
313.2	135	129	0.65 ± 0.08	1.29 ± 0.16	1.46 ± 0.18
	287	281	0.69 ± 0.09	1.37 ± 0.17	1.55 ± 0.19
	481	475	0.69 ± 0.09	1.37 ± 0.17	1.55 ± 0.19
	899	893	0.66 ± 0.17	1.32 ± 0.33	1.50 ± 0.37
	1186	1180	0.71 ± 0.13	1.41 ± 0.26	1.60 ± 0.29
323.2	155	144	0.62 ± 0.08	1.24 ± 0.16	1.40 ± 0.18
	313	302	0.66 ± 0.09	1.31 ± 0.17	1.48 ± 0.19
	509	498	0.67 ± 0.09	1.33 ± 0.17	1.50 ± 0.19
	937	926	0.65 ± 0.17	1.30 ± 0.33	1.47 ± 0.37
	1245	1234	0.67 ± 0.13	1.34 ± 0.26	1.51 ± 0.29
333.2	179	161	0.59 ± 0.08	1.18 ± 0.16	1.33 ± 0.19
	341	323	0.63 ± 0.09	1.26 ± 0.17	1.42 ± 0.19
	981	963	0.62 ± 0.17	1.24 ± 0.33	1.40 ± 0.37
	1304	1286	0.64 ± 0.13	1.27 ± 0.26	1.43 ± 0.29

[a]Standard uncertainty in temperature u(T) = 0.10 K

[b]Standard uncertainty in total pressure u(p) = 0.005·P
